# Targeting of p21-Activated Kinase 4 Radiosensitizes Glioblastoma Cells via Impaired DNA Repair

**DOI:** 10.3390/cells11142133

**Published:** 2022-07-06

**Authors:** Leon J. Blankenstein, Nils Cordes, Leoni A. Kunz-Schughart, Anne Vehlow

**Affiliations:** 1 OncoRay—National Center for Radiation Research in Oncology, Faculty of Medicine Carl Gustav Carus, Technische Universität Dresden, Fetscherstr. 74, PF 41, 01307 Dresden, Germany; leon.blankenstein@tu-dresden.de (L.J.B.); nils.cordes@oncoray.de (N.C.); leoni.kunz-schughart@oncoray.de (L.A.K.-S.); 2 National Center for Tumor Diseases, Partner Site Dresden: German Cancer Research Center, Im Neuenheimer Feld 280, 69120 Heidelberg, Germany; 3 Department of Radiotherapy and Radiation Oncology, University Hospital Carl Gustav Carus, Technische Universität Dresden, Fetscherstr. 74, PF 50, 01307 Dresden, Germany; 4 Helmholtz-Zentrum Dresden—Rossendorf, Institute of Radiooncology—OncoRay, Bautzner Landstr. 400, 01328 Dresden, Germany; 5 German Cancer Consortium (DKTK), Partner Site Dresden, and German Cancer Research Center (DKFZ), Im Neuenheimer Feld 280, 69192 Heidelberg, Germany

**Keywords:** glioblastoma, p21-activated kinase 4, radiosensitivity, DNA repair

## Abstract

Glioblastoma is a devastating malignant disease with poor patient overall survival. Strong invasiveness and resistance to radiochemotherapy have challenged the identification of molecular targets that can finally improve treatment outcomes. This study evaluates the influence of all six known p21-activated kinase (PAK) protein family members on the invasion capacity and radio-response of glioblastoma cells by employing a siRNA-based screen. In a panel of human glioblastoma cell models, we identified PAK4 as the main PAK isoform regulating invasion and clonogenic survival upon irradiation and demonstrated the radiosensitizing potential of PAK4 inhibition. Mechanistically, we show that PAK4 depletion and pharmacological inhibition enhanced the number of irradiation-induced DNA double-strand breaks and reduced the expression levels of various DNA repair proteins. In conclusion, our data suggest PAK4 as a putative target for radiosensitization and impairing DNA repair in glioblastoma, deserving further scrutiny in extended combinatorial treatment testing.

## 1. Introduction

Glioblastomas are the most common malignant primary brain tumors in adults [1]. Despite a multimodal therapy approach consisting of surgery and radio- and chemotherapy, glioblastoma patients suffer from disease relapse and progression beyond the initial tumor treatment, leading to a median overall survival of only 1 to 2 years [2,3]. Particular genetic and epigenetic alterations, strongly heterogenic malignant cell clones, and resistance to radio- and chemotherapy are causal to the poor prognosis [4,5,6,7]. Furthermore, infiltration of glioblastoma cells to brain areas close to the primary site makes a complete resection impossible and results in the destruction of normal brain structures [8,9]. Therefore, many efforts have been made to unravel the mechanisms triggering glioblastoma evasion of therapy-induced cell death to identify new therapeutic targets and expand the life expectancy of patients. Targeting immunogenic or angiogenic pathways are two fields of intense research [10,11,12,13]. Likewise, DNA damage repair pathways are increasingly recognized in providing possible targets for multimodal therapy for glioblastoma [14,15,16,17].

DNA damage repair is modulated by a plethora of cell signaling mechanisms, including crosstalk of membrane-bound receptors, cytoplasmic kinases, and the cellular cytoskeleton [18,19]. PAKs (p21-activated kinases) are a protein family linked to cell mobilization, proliferation, and cell survival [20]. Based on structural and functional aspects, the six known family members are categorized into group I PAKs, i.e., PAK1–3, and group II PAKs, comprised of PAK4–6 [21,22]. Increasing evidence suggests a key role of PAKs and associated signaling events in tumor development, progression, and therapy resistance [23]. Studies in various cancer entities underline that PAKs can function as drivers of epithelial-to-mesenchymal transition (EMT), cancer growth, and resistance to chemotherapy or small molecule inhibitors [20,24,25,26]. In glioblastoma, PAK1 phosphorylation in the cytoplasm and its expression level correlate with patient prognosis [27,28]. Furthermore, the inhibition of PAK5 reduces glioblastoma cell tumorigenicity [29]. PAK4 appears to be functionally associated with the migration, invasion, and EMT of glioblastoma cells and is involved in preventing senescence-linked cell cycle arrest and proliferation [30,31,32,33].

DNA double-strand breaks (DSBs) are the most complex DNA damage induced by radiation and chemotherapy, and they substantially determine the success of currently available treatment options [34,35]. A highly effective DNA repair system repairs DSBs with two main mechanisms: non-homologous end joining (NHEJ) and homologous recombination (HR) [36]. Malfunctions of these mechanisms in malignant cells, such as altered activity and expression of key DNA repair proteins, can lead to improved DNA repair and the resistance of cancer cells to genotoxic agents [11,37]. In glioblastoma, oncogenic mutations in key signaling networks, such as p53 and retinoblastoma signaling, as well as methylation of the O^6^-methylguanine-DNA methyltransferase (MGMT) promoter, are frequently associated with DNA repair pathway alterations [38,39]. Therefore, targeting DNA repair mechanisms may provide a therapeutic advantage for glioblastoma patients, and pharmacological inhibitors blocking key DNA repair enzymes are currently being tested in clinical trials (www.clinicaltrials.gov, accessed on 1 June 2022).

Overall, the preexisting data imply PAK family members’ individual or integrated impact on glioblastoma cell behavior and therapy outcome. However, whether this is also true for the response to radiotherapy and linked to irradiation-induced DNA damage repair remains ambiguous. We, therefore, evaluated the influence of the PAK protein family on the radiosensitivity, in parallel with the invasive capacity, of glioblastoma cells by using a siRNA knockdown screen targeting all PAK isoforms. This approach identified PAK4 as a critical determinant, as PAK4 knockdown and its pharmacological inhibition reduced both clonogenic radiation survival and invasion in a panel of human glioblastoma cell line models. We further demonstrated that the radiosensitizing potential of PAK4 depletion is associated with an increase in DSBs after irradiation. Analysis of the underlying molecular mechanism upon PAK4 targeting revealed reduced expression levels of key DNA repair proteins such as the ataxia telangiectasia-mutated (ATM) serine/threonine kinase, the DNA-dependent protein kinase (DNA-PK), poly(ADP-ribose)polymerase 1 (PARP1), and the Rad51 recombinase. Overall, our study provides functional evidence for a PAK4-dependent modulation of DNA repair mechanisms leading to radioresistance in glioblastoma cells.

## 2. Materials and Methods

### 2.1. Antibodies

Antibodies for Western blot and immunofluorescence staining were purchased as follows: PAK1, PAK2, PAK3, PAK4, phospho-PAK4/5/6 (S474, S602, S560), PARP1, ATM, DNA-PK, PAK5, phospho-DNA PKs (S2056), and PAK6 (all Cell Signaling Technology, Frankfurt am Main, Germany); β-actin (Sigma-Aldrich); phospho-ATM (S1981) (Rockland Immunochemicals, Gilbertsville, PA, USA); Rad51 and γH2AX (both Merck Millipore, Burlington, MA, USA), 53BP1 (Novus Biologicals, Wiesbaden Nordenstadt, Germany); goat anti-mouse AlexaFluor488 and goat anti-rabbit AlexaFluor546 (both Life Technologies, Carlsbad, CA, USA); donkey anti-rabbit HRP and sheep anti-mouse HRP (both GE Healthcare, Chicago, IL, USA).

### 2.2. Cell Culture

U251MG and LN229 human glioblastoma cell lines were obtained from the American Type Culture Collection (ATCC); U343MG, DD-T4 and DD-HT7606 cells were kindly provided by A. Temme (Technische Universität Dresden, Dresden, Germany). U343MG were cultured in basal medium Eagle (BME, Thermo Fisher Scientific, Waltham, MA, USA), supplemented with 10% FBS (PAN-Biotech, Aidenbach, Germany), 1% NEAA (Thermo Fisher Scientific), 10 mM 4-(2-hydroxyethyl)-1-piperazineethanesulfonic acid (HEPES, Sigma-Aldrich, Taufkirchen, Germany), and 2 mM L-Glutamine (Sigma-Aldrich) at 37 °C with 5% CO_2_ and a humidified atmosphere. All other cell lines were grown in Dulbecco’s modified Eagle’s medium (DMEM, Thermo Fisher Scientific), supplemented with 10% FBS and 1% non-essential amino acids (Thermo Fisher Scientific) at 37 °C and 8.5% CO_2_. All cell lines were authenticated by short tandem repeat DNA profiling and tested mycoplasma-negative.

### 2.3. Radiation Exposure

Irradiation was applied at room temperature using single doses of 2, 4, and 6 Gy 200 kV X-rays (Yxlon Y.TU 320; Yxlon, Kopenhagen, Denmark) filtered with 0.5 mm copper. The approximate dose rate was 1.3 Gy/min at 20 mA. The absorbed dose was routinely monitored using a Duplex dosimeter before the exposure of the samples (PTW, Freiburg, Germany).

### 2.4. Small Interfering RNA (siRNA) Transfection

SiRNA transfection was accomplished as previously described [40]. In brief, 24 h after plating, cells were treated with 20 nM siRNA diluted with oligofectamine (Invitrogen). After 48 h, cells were subjected to colony formation assay, invasion assay, and Western blot analysis. The following small interfering RNAs were purchased as indicated: 5′-CCAAGAAAGAGCTGATTATTtt-3′ (PAK1#1), 5′-GAAGGACCGAUUUUACCGAtt-3′ (PAK1#2), 5′-GAACUGAUCAUUAACGAGAtt-3′ (PAK2#1), 5′-CAGAGGUGGUUACACGGAAtt-3′ (PAK2#2), 5′-CAACCCAAGAAGGAAUUAAtt-3′ (PAK3#1), 5′-TAGCAGCACATCAGTCGAATA-3′ (PAK3#2), 5′-GGUGGAUUACGAUCGAGCAtt-3′ (PAK5#1), 5′-GGAUUACCACCAU-GACAAUtt-3′ (PAK5#2) (all from Thermo Fisher Scientific), 5′-GGGUGAAGCUGUCAGACUUtt-3′ (PAK4#1), 5′-ACUAAGAGGUGAACAUGUAtt-3′ (PAK4#2), 5′-GCAGCUAUAUGAAUGUUGUtt-3′ (Control) (all from Eurofins MWG), 5′-GAAGUGAUCUCCAGGUCUU-3′ (PAK6#1) and 5′-CCACCGACCCAGACAUGUA-3′ (PAK6#2) (all from Dharmacon).

### 2.5. Colony Formation Assay

Clonogenic survival was analyzed as previously described [41] by plating single cells onto collagen-type-I-coated 6-well plates (1 µg/cm^2^, BD Biosciences, Heidelberg, Germany). Cells were irradiated with 2, 4, and 6 Gy single X-ray doses 24 h after plating. After cell-line-specific incubation times, cells were fixed with 80% EtOH and then stained with Coomassie blue (Merck Millipore). The incubation times were as follows: 7 days for LN229 and 10 days for all other cell line models. To evaluate clonogenic survival, colonies with more than 50 cells were manually counted using a Stemi 2000 stereo microscope (Carl Zeiss Microscopy GmbH, Jena, Germany).

### 2.6. Cell Proliferation Assay

Cell proliferation was assessed by seeding 1000 cells in 24-well tissue culture plates. For cell counting, cells were trypsinized, diluted in cell culture medium, and counted using a Neubauer chamber 5 days after seeding.

### 2.7. 3D Spheroid Invasion Assay

Glioblastoma spheroids were generated by culturing 10,000 cells in 96-well U-bottom plates coated with 1% agarose. After 48–72 h, spheroids were transferred into a 3D type I collagen matrix (3 mg/mL, BD Biosciences). Images of cells migrating out of the spheroid were acquired at 72 h of invasion using an Axiovert 200 microscope (Carl Zeiss Microscopy GmbH), and the distance of invading cells was measured as described [41].

### 2.8. Total Protein Extracts and Western Blot

Cells were lysed in 1× RIPA buffer consisting of 50 mM Tris-HCl (pH 7.4, AppliChem, Darmstadt, Germany), 1% Nonidet-P40 (Sigma-Aldrich), 0.25% sodium deoxycholate, 150 mM NaCl, 1 mM EDTA, 1 mM NaVO_4_, 2 mM NaF (all Sigma-Aldrich), and Complete^TM^ protease inhibitor cocktail (Roche, Basel, Switzerland). The measurement of protein concentration was achieved by using a BCA assay kit (Thermo Fisher Scientific). Proteins were transferred onto nitrocellulose membranes (GE Healthcare) after SDS-PAGE. Detection of specific proteins was accomplished after blocking by incubating the membranes with specific primary antibodies and horseradish peroxidase-conjugated secondary antibodies. Bands were visualized with an enhanced chemiluminescent reagent (ECL, GE Healthcare) and detected using a Chemi Doc Western Blot Imager (Bio-Rad, Hercules, CA, USA).

### 2.9. Analysis of DNA DSB

To determine the DNA DSB caused by irradiation, cells were fixed before and 24 h after 6 Gy X-ray irradiation using 3% formaldehyde (Sigma-Aldrich). Permeabilization of cells was achieved by incubation with 0.25% Triton-X-100/PBS (Sigma-Aldrich). After blocking with 1% BSA/PBS (Sigma-Aldrich), cells were incubated with primary antibodies specific for 53BP1 and γH2AX as well as secondary antibodies conjugated with AlexaFluor488 and AlexaFluor546. Cells were mounted with ProLong Diamond Antifade Mountant with DAPI counterstain (Thermo Fisher Scientific). Foci double-positive for γH2AX and 53BP1 were manually counted for DSB quantification in nuclei using an AxioImager M1 (Carl Zeiss Microscopy GmbH).

### 2.10. Immunofluorescence Staining

To analyze the localization of the different PAK isoforms, glioblastoma cells were fixed with 3% formaldehyde, permeabilized with 0.25% Triton-X-100/PBS, blocked with 1% BSA/PBS, and stained with specific primary and AlexaFluor488-conjugated secondary antibodies. F-actin was stained with AlexaFluor594-Phalloidin (Life Technologies). Cells were mounted using ProLong Diamant Antifade Mountant with DAPI, and images were acquired using an AxioImager M1 (Carl Zeiss Microscopy GmbH).

### 2.11. Statistical Analysis

Means +/− standard deviations (SDs) were calculated from at least three independent biological experiments (indicated as N). As indicated, statistical evaluation was determined with a two-sided Student’s *t*-test using Excel 2016 (Microsoft, Redmont, WA, USA) or one-way ANOVA with Dunnett’s or Bonferroni’s post-hoc test using PRISM 8 (GraphPad Software, San Diego, CA, USA). A *p*-value of less than 0.05 was considered statistically significant.

## 3. Results

### 3.1. PAK Isoforms Differentially Contribute to Glioblastoma Cell Radiosensitivity and Invasion

**Figure 1:**
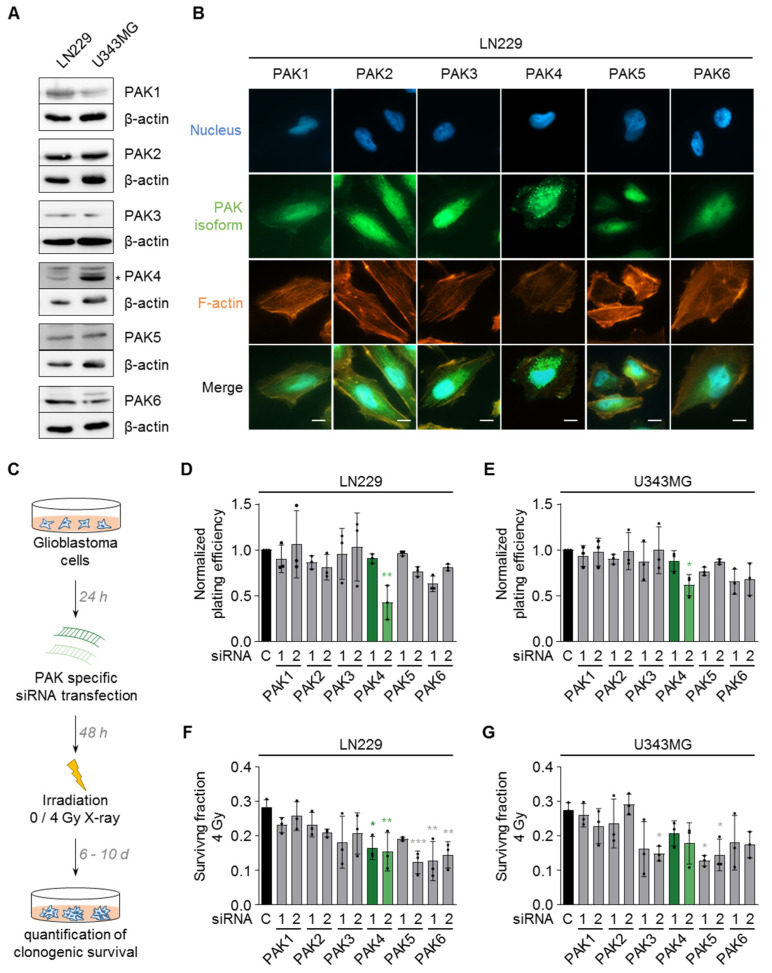
PAK isoforms differentially regulate glioblastoma cell radiosensitivity. (**A**) Representative Western blot images of the expression of the indicated PAK isoforms in LN229 and U343MG cell lines. β-actin served as loading control. (**B**) Representative immunofluorescence images of the nucleus (DAPI), the indicated PAK isoforms, and F-actin (Phalloidin) in LN229 cells. Scale bar: 5 µm. (**C**) Workflow of the colony formation assay for the quantification of clonogenic survival. (**D**,**E**) Normalized plating efficiency (basal clonogenic survival) of (**D**) LN229 and (**E**) U343MG cells upon knockdown of the different PAK isoforms. (**F**,**G**) Surviving fraction of (**F**) LN229 and (**G**) U343MG cells upon knockdown of the different PAK isoforms with and without 4 Gy X-ray irradiation. (**D**–**G**) Data show means +/– SDs (N = 3 independent experiments, one-way ANOVA, Dunnett’s post-hoc test, * *p* < 0.05, ** *p* < 0.01, *** *p* < 0.001).

First, we evaluated the expression and localization of the six known PAK isoforms (PAK1–PAK6) in two human glioblastoma cell lines, LN229 and U343MG. Western blot analysis of these cell lines demonstrated a cell-line-dependent expression of all PAK isoforms (Figure 1A). Immunofluorescence stainings revealed a subcellular distribution of the different PAK isoforms to the cytoplasm as well as the nucleus (Figure 1B and Figure S1A), which is in line with recent studies [42,43,44].

To elucidate their function for glioblastoma cell radiosensitivity, we performed a siRNA-based knockdown screen targeting all PAK isoforms in LN229 and U343MG cells (Figure S1B,C) and quantified clonogenic survival without and in combination with irradiation (Figure 1C). Under non-irradiated conditions, PAK4 knockdown decreased clonogenic survival in both cell lines relative to the control and the other PAK isoforms (Figure 1D,E). In contrast, we observed cell-line-dependent effects upon knockdown of the different PAK isoforms in combination with 4 Gy X-ray irradiation. In LN229 cells, knockdown of the group II PAK family members PAK4, PAK5, and PAK6 induced radiosensitization, while we observed radiosensitization upon PAK3 and PAK5 knockdown in U343MG cells (Figure 1F,G).

**Figure 2:**
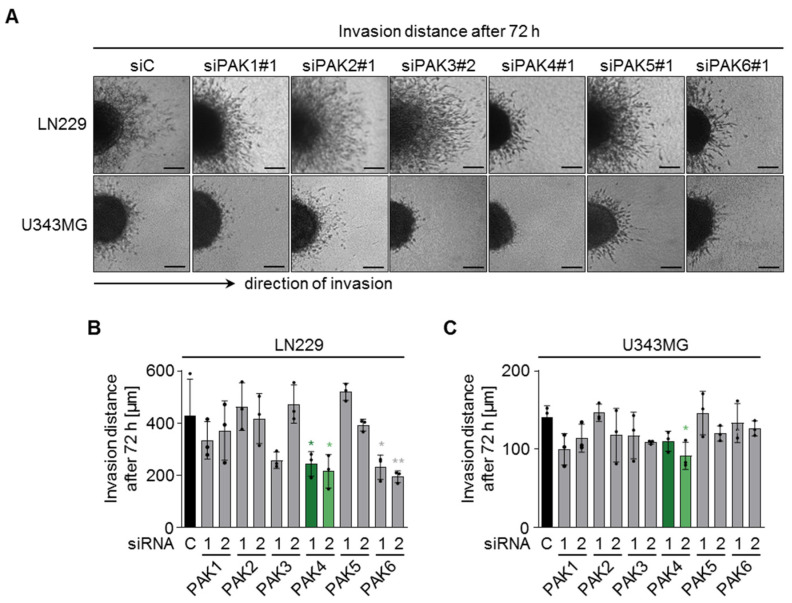
PAK isoforms differentially regulate glioblastoma cell invasion. (**A**) Representative images of LN229 and U343MG spheroids 72 h after transfer to a three-dimensional collagen type I matrix. Scale bar: 200 µm. (**B**) Quantification of invasion distance of LN229 cells and (**C**) U343MG cells upon knockdown of the different PAK isoforms and 72 h of invasion. (**B**,**C**) Data show means +/− SDs (N = 3 independent experiments, ∑n = 15 spheroids per treatment condition, one-way ANOVA, Dunnett’s post-hoc test, * *p* < 0.05, ** *p* < 0.01).

Next, we investigated the impact of the different PAK isoforms on LN229 and U343MG cell invasion in a three-dimensional collagen type-1 matrix upon siRNA-mediated knockdown of all PAK isoforms. Knockdown of PAK4 significantly reduced the 3D invasion distance after 72 h in both cell lines, despite their different basal invasion capacities (Figure 2A–C). Furthermore, PAK6 decreased 3D invasion in LN229 cells (Figure 2A–C).

Taken together, our results show cell-line-specific functions of the different PAK isoforms in the glioblastoma cell response to ionizing irradiation and in their invasive behavior. Of all six isoforms, PAK4 appears to play the most important role in the radiosensitivity and invasion of glioblastoma cells.

### 3.2. Glioblastoma Cell Radiosensitivity and Proliferation Depends on PAK4

**Figure 3:**
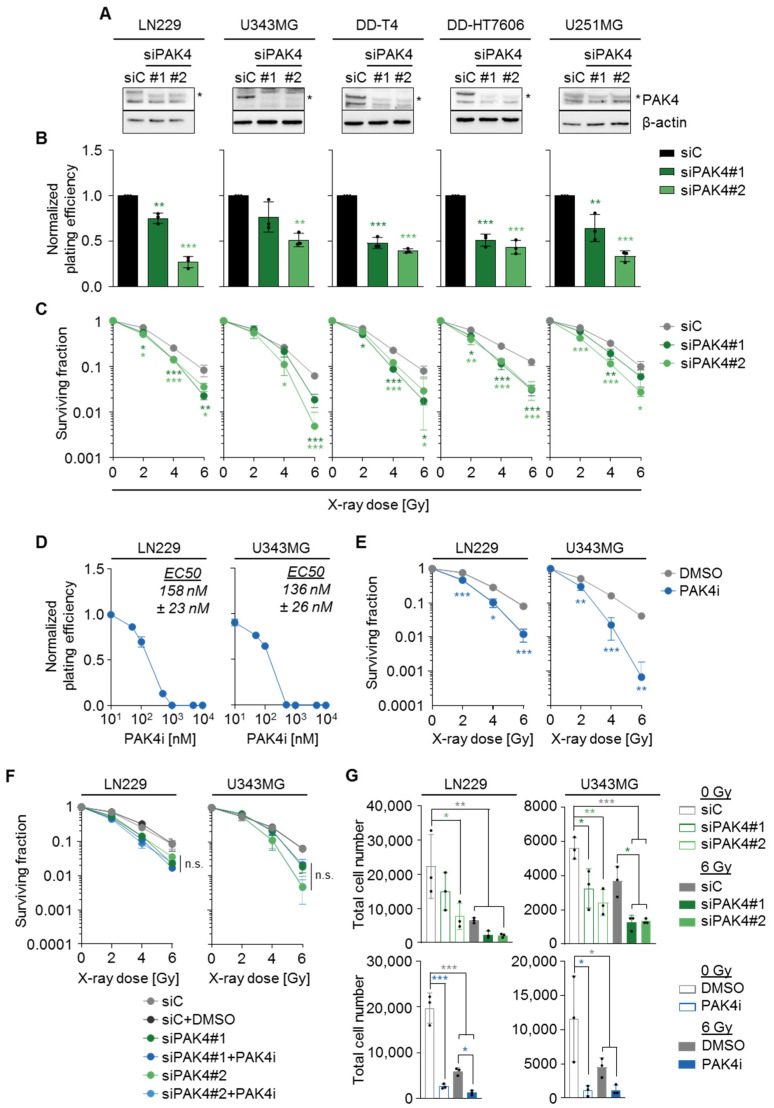
PAK4 targeting radiosensitizes glioblastoma cells. (**A**) Representative Western blot images showing the efficiency of siRNA-mediated knockdown of PAK4 in whole-cell lysates of LN229, U343MG, DD-T4, DD-HT7606, and U251MG cells. β-actin served as loading control. (**B**) Normalized plating efficiency of indicated glioblastoma cells upon siRNA-mediated knockdown of PAK4. (**C**) Surviving fraction of indicated glioblastoma cell lines after siRNA-mediated knockdown of PAK4 combined with X-ray irradiation (0–6 Gy). (**B**,**C**) Data show means +/− SD (N = 3 independent experiments, one-way ANOVA, Dunnett’s post-hoc test, * *p* < 0.05, ** *p* < 0.01, *** *p* < 0.001) (**D**) Normalized plating efficiency of LN229 and U343MG cells treated with increasing doses of the PAK4 inhibitor PF-3758309. EC50 values were calculated based on the dose–response curves. (**E**) Surviving fraction of LN229 and U343MG glioblastoma cells after treatment with PAK4i EC50 in combination with X-ray irradiation. Data show means +/− SD (N = 3 independent experiments, *t*-test, * *p* < 0.05, ** *p* < 0.01, *** *p* < 0.001) (**F**) Surviving fraction of LN229 and U343MG cells after treatment with siPAK4 and siPAK4 plus PAK4i combined with X-ray irradiation. (**G**) Total cell number indicating proliferation of LN229 and U343MG cells 5 days after siRNA-mediated knockdown of PAK4 (upper panels) and treatment with PAK4i EC50 (lower panels) in combination with X-ray irradiation. (**F**,**G**) Data show means +/− SDs (N = 3 independent experiments, one-way ANOVA, Bonferroni’s post-hoc test, * *p* < 0.05, ** *p* < 0.01, *** *p* < 0.001, n.s. not significant).

Among the group II PAK isoforms, PAK4 is linked to glioblastoma cell proliferation, senescence, and migration [30,33], corroborating the connection to glioblastoma therapy resistance. We focused our further analyses on potential mechanisms linking PAK4 to the glioblastoma radiation response. To validate PAK4 as a central determinant of clonogenic radiation survival, we extended our knockdown analysis to a panel of five glioblastoma cell lines (Figure 3A). Our results revealed significantly decreased basal clonogenic survival and demonstrated radiosensitization upon PAK4 knockdown in all tested cell line models (Figure 3B,C). Additionally, we applied the pharmacological PAK4 inhibitor PF-3758309 (PAK4i) [45] to LN229 and U343MG cells to confirm the link between PAK4 and glioblastoma cell radiosensitivity (Figure 3D,E). To examine the specificity of PAK4i for clonogenic radiation survival, we treated LN229 and U343MG cells with PAK4i EC50 upon PAK4 knockdown. In these cells, PAK4i failed to induce additional radiosensitization, confirming the specific molecular efficacy of the PAK4i (Figure 3F). In addition to clonogenic survival, we analyzed cell proliferation upon PAK4 silencing and PAK4i combined with X-ray irradiation and found severely decreased proliferative capacities in both cell lines 5 days after plating. Hence, our findings indicate an essential function of PAK4 in regulating glioblastoma radiosensitivity and proliferation.

### 3.3. Disruption of PAK4 Signaling Causes Accumulation of DNA Damage

**Figure 4:**
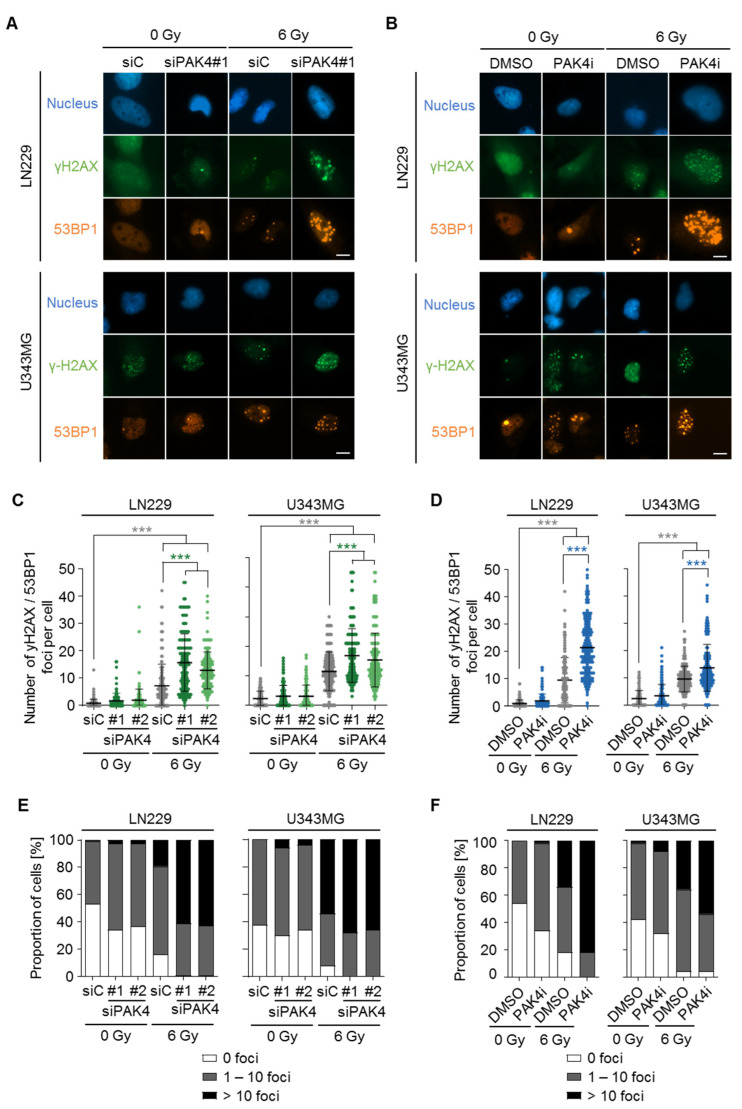
PAK4 depletion triggers an accumulation of DNA double-strand breaks. (**A**,**B**) Representative immunofluorescence images of the nucleus (DAPI), γH2AX, and 53BP1 in LN229 and U343MG cells upon treatment with (**A**) siPAK4#1 and (**B**) PAK4i EC50 without or in combination with 6 Gy X-ray irradiation. Scale bar: 5 µm. (**C**,**D**) Quantification of γH2AX/53BP1 foci in LN229 and U343MG cells upon (**C**) siRNA-mediated depletion of PAK4 and (**D**) PAK4i treatment in unirradiated and 6 Gy X-ray irradiated cells. (**E**,**F**) Proportion of cells with indicated numbers of γH2AX/53BP1 foci upon (**E**) depletion of PAK4 and (**F**) PAK4i treatment before and after 6 Gy X-ray irradiation. (**C**–**F**) Data show means +/− SDs (N = 3 independent experiments, ∑n = 150 cells per treatment condition), one-way ANOVA, Bonferroni’s post-hoc test, *** *p* < 0.001).

Next, we assessed the number of DSBs upon PAK4 targeting, as their accumulation links to therapy sensitization. We stained LN229 and U343MG cells treated with either siPAK4 or PAK4i without and in combination with 6 Gy X-ray irradiation for γH2AX and 53BP1, two typical markers of DSBs (Figure 4A,B). In line with the survival data, both LN229 and U343MG cells showed slightly higher amounts of residual DSBs upon knockdown and inhibition of PAK4 relative to controls (Figure 4C,D). Upon X-ray irradiation, PAK4 targeting significantly increased γH2AX/53BP1 double-positive foci in both cell lines compared with non-irradiated controls (Figure 4C,D) and substantially increased the proportion of cells with foci (Figure 4E,F). Taken together, these results suggest a critical role of PAK4 in mediating the repair of radiogenic DNA damage.

### 3.4. PAK4 Inactivation Modulates DNA Repair

**Figure 5:**
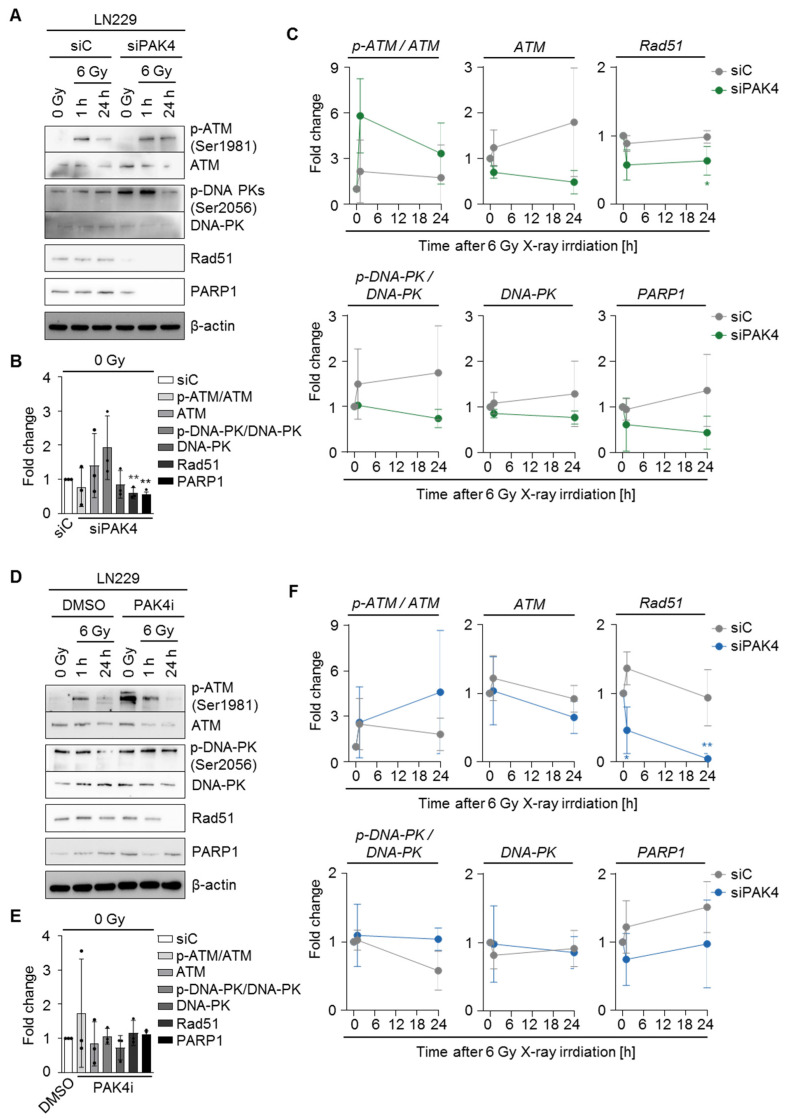
PAK4 regulates the expression of key DNA repair proteins. (**A**) Representative Western blot images of the phosphorylation and expression of the indicated DNA repair proteins in dependence on siC or siPAK4#1 in unirradiated LN229 cells and after 1 and 24 h of 6 Gy X-ray irradiation. (**B**,**C**) Quantification of the phosphorylation and expression levels of the indicated DNA repair proteins after siPAK4#1 treatment in (**C**) unirradiated and (**D**) irradiated LN229 cells. Values are depicted as fold changes of siC. (**D**) Western blot analysis of the phosphorylation and expression of the indicated DNA repair proteins in dependence on DMSO and PAK4i in unirradiated LN229 cells and after 1 and 24 h of 6 Gy X-ray irradiation. (**E**,**F**) Quantification of the phosphorylation and expression levels of the indicated DNA repair proteins after PAK4i treatment in (**E**) unirradiated and (**F**) irradiated LN229 cells. Values are depicted as fold changes of DMSO. (**B**,**C**,**E**,**F**) Data show means +/− SDs (N = 3 independent experiments, *t*-test, * *p* < 0.05, ** *p* < 0.01).

Based on the enhanced DSB numbers upon PAK4 targeting, we tested the phosphorylation and expression status of key DNA repair proteins ATM, DNA-PK, Rad51, and PARP1 upon PAK4 depletion and PAK4 inhibition combined with irradiation using Western blot analysis in LN229 cells (Figure 5A,D). In unirradiated cells, PAK4 depletion enhanced the expression of ATM and the phosphorylation of DNA-PK (Ser2056) while reducing Rad51 and PARP1 levels (Figure 5A,B). After PAK4 knockdown and irradiation, we observed an increased phosphorylation of ATM Ser1981 that was associated with diminished phospho-DNA-PK (Ser2056) and ATM, DNA-PK, Rad51, and PARP1 levels (Figure 5A,C). PAK4 inhibitor administration lacked changes in phosphorylation or expression under unirradiated conditions (Figure 5D,E) but elevated ATM S1981 phosphorylation and reduced ATM, Rad51, and PARP1 expression levels, similar to the genetic deactivation (Figure 5D,F). Together with the enhanced number of foci after PAK4 targeting, these data mechanistically link PAK4 to the repair of radiogenic DSBs.

## 4. Discussion

For almost 15 years, surgery followed by radiochemotherapy with temozolomide has been the standard of care for glioblastoma patients [2]. Since this has proven to be insufficient to achieve satisfactory survival rates, molecular-targeted attempts against key proteins involved in cancer development, progression, survival, and motility have been undertaken to improve patient prognosis. In this study, we investigated the function of the PAK protein family on the radioresistance and invasion of glioblastoma cells and examined the underlying mechanism. We show that (i) PAK4 critically contributes to the radiosensitivity and invasion of glioblastoma cells, (ii) PAK4 knockdown and pharmacological targeting elicit radiosensitization along with increased DNA damage, and (iii) PAK4 modulates the expression and phosphorylation of the key DNA repair proteins ATM, DNA-PK, Rad51, and PARP1.

In a siRNA-based screen targeting all PAK isoforms, we identified PAK4 to mediate both the invasion as well as radioresistance of glioblastoma cells. Our study is the first to demonstrate the potential of PAK4 targeting for glioblastoma radiosensitization. In a study from 2010 on prostate cancer, PAK6 depletion also enhanced cellular radiosensitivity [46], suggesting an effect of this protein on therapy resistance. In line with these data, we now provide evidence of a general function of group II PAK for the radiosensitivity of glioblastoma cells. In addition, our data on PAK4-mediated collagen invasion are in line with recent studies suggesting a prominent role of PAK4 in regulating the progression of glioblastoma [30,31,32] and metastasis in other cancer entities [47,48,49]. Hence, our results add a further facet to the role of PAK4 in directing cancer cell motility. In terms of the clinical feasibility of PAK4 targeting, earlier studies using the PAK4 inhibitor PF-3758309 in patients with advanced solid tumors failed due to pharmacokinetic difficulties. However, new PAK4 inhibitors are under development or in clinical testing [50] (www.clinicaltrial.gov, accessed on 1 June 2022).

In immunofluorescence stainings, we observed that PAK4, among other isoforms, localizes to the cell nucleus and cytoplasm. However, if and how the nuclear and cytoplasmic localizations of the different PAK isoforms link to overall survival and therapy response is only beginning to emerge. Earlier studies identified an association between high PAK1 expression, in general, particularly of phosphorylated cytoplasmic PAK1, and poor prognoses in glioblastoma patients [27,28]. In contrast, nuclear PAK1 binds to chromatin and may be involved in gene transcription [42,44] and DNA damage response [51,52]. PAK4 localizes to the cytoplasm and nucleus in breast cancer cells, and its expression correlates with an advanced tumor stage [53]. Similar results have been shown in glioma, where PAK4 expression intensifies with an increasing pathological grade [33]. Together, these data suggest specific regulatory functions for nuclear and cytoplasmic PAK isoforms, a fact that warrants further investigation.

Here, we report a relationship between PAK4 and the cellular DNA damage response in glioblastoma cells, as PAK4 inhibition increases residual DSBs. These findings are in line with recent reports linking PAK4 to the expression of different DNA repair proteins in Morbus Waldenström and multiple myeloma [54,55]. Along with these findings, we demonstrate a reduction of ATM, DNA-PK, PARP1, and RAD51 expression and DNA-PK phosphorylation levels and an increase in ATM after targeting PAK4 in combination with X-ray irradiation. These observed changes suggest that PAK4 carries out a more general function for preserving DNA repair via the HR and NHEJ pathways in response to radiation-induced DNA damage. How PAK4 links to the expression of these proteins and the repair of DSBs remains unknown. One possible mechanism might be attributed to the regulatory function of PAK4 for cell cycle progression [56], which directly links to the functionality of the different DNA repair pathways mirrored by the cell-cycle-dependent expression of DNA repair proteins [57]. On the other hand, PAK4 activates the transcription factor nuclear factor kappa-light-chain-enhancer of activated B-cells (NF-κB), which transcribes DNA repair genes and participates in DSB repair [58,59,60]. Furthermore, PAK4 phosphorylates Runt-related transcription factor 1 (RUNX1), a regulator of the DNA damage response in breast cancer cells [61,62]. In glioma, a nuclear association between PAK4 and the peroxisome proliferator-activated receptor gamma (PPARγ) regulates epithelial-to-mesenchymal transition [32], while PPARγ itself mediates the expression and function of DNA damage response genes [63,64]. Overall, these studies indicate that PAK4 may exert its effects via promoting the transcription of DNA repair genes through various mechanisms, warranting further investigation.

## 5. Conclusions

In conclusion, we demonstrate a so-far undescribed function of PAK4 in regulating glioblastoma cell radiosensitivity through the modulation of DNA damage repair mechanisms. Hence, our data suggest PAK4 as a potential molecular target to overcome glioblastoma radioresistance. Future in vitro and in vivo studies are necessary to validate the effectiveness of PAK4 inhibition as a therapeutic approach.

## Data Availability

Not applicable.

## Supplementary Materials

The following supporting information can be downloaded at: https://www.mdpi.com/article/10.3390/cells11142133/s1, Figure S1: Localization of PAK isoforms.
