# Supplementary figures and images for: Targeting of p21-Activated Kinase 4 Radiosensitizes Glioblastoma Cells via Impaired DNA Repair
Source: Cells. 2022 Jul 6;11(14):2133. doi: 10.3390/cells11142133 (PMC9316146; doi:10.3390/cells11142133)

**A**

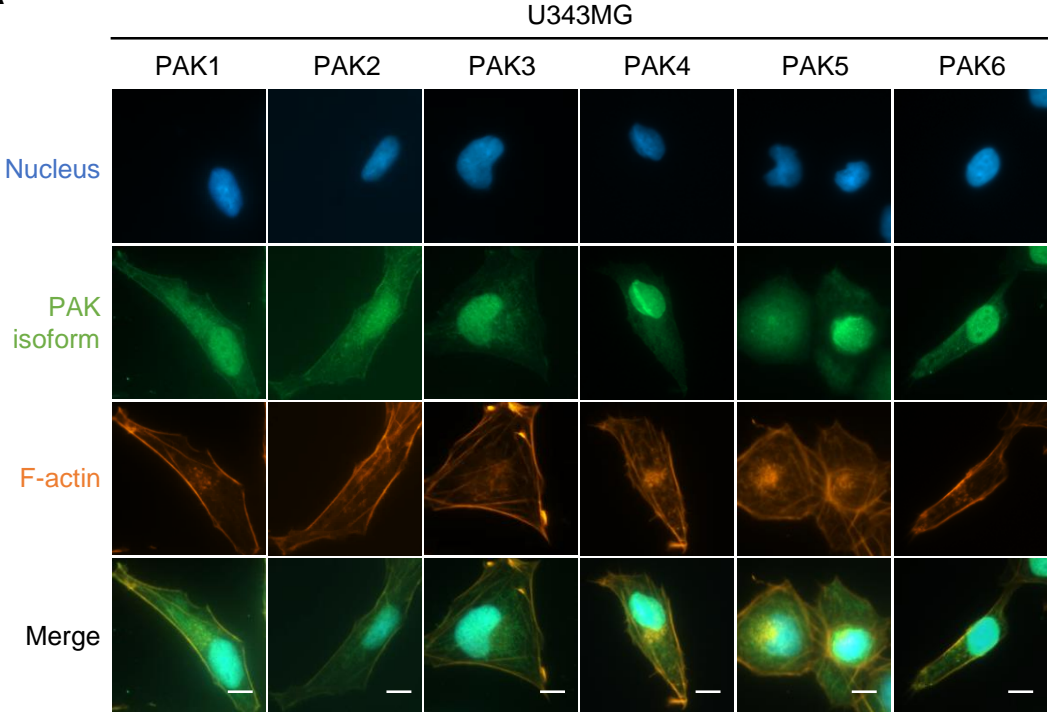

**B**

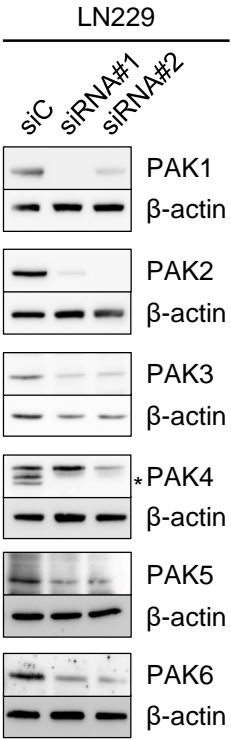

**C**

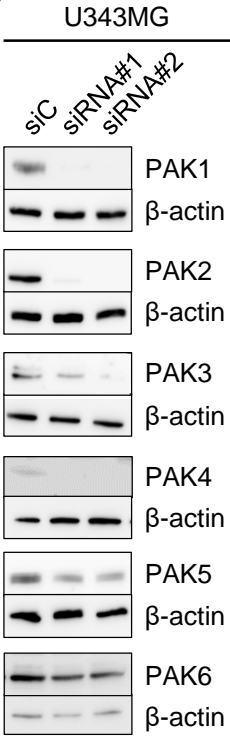

**Supplementary Figure S1 – Blankenstein et al.**

Supplement: Supplementary file 1 [file cells-11-02133-s001.zip › cells-1796425-supplementary.pdf]
